# Emergence of Macrolide-Resistant *Bordetella pertussis*, Peru, 2025

**DOI:** 10.3201/eid3210.251477

**Published:** 2026-10

**Authors:** Eduardo Juscamayta-López, Faviola Valdivia, Carmen Rodríguez-Cueva, Helen Horna, Yaneth Quispe, María Pía Soto, Segundo Torres, Victor Fiestas Solórzano, Martin Yagui, Vlademir Vicente Cantarelli, Efrain Montilla-Escudero, Víctor Silva, Ana Raquel Da Costa

**Affiliations:** Instituto Nacional de Salud, Lima, Peru (E. Juscamayta-López, F. Valdivia, C. Rodríguez-Cueva, H. Horna, Y. Quispe, M. Pía Soto, S. Torres, V. Fiestas Solórzano, M. Yagui); American Society for Microbiology, Washington DC, USA (V. Vicente Cantarelli, E. Montilla-Escudero, V. Silva, A. Raquel Da Costa).

**Keywords:** *Bordetella pertussis*, pertussis, bacteria, respiratory infections, whooping cough, pertactin, macrolides, antimicrobial resistance, macrolide resistance, vaccine-preventable diseases, molecular epidemiology, Peru

## Abstract

We report the emergence of macrolide-resistant Bordetella pertussis during a pertussis outbreak in Peru. Among 68 cases, 31% carried the A2047G gene mutation, conferring resistance to macrolides. Whole-genome sequencing revealed 2 genetically distinct groups, indicating multiple introductions into Peru. Our findings support strengthening surveillance for macrolide-resistant pertussis to inform control strategies.

The bacterium *Bordetella pertussis* is the causative agent of pertussis, a highly contagious respiratory infection, which poses the greatest risk for severe illness and death in infants (*1*). Despite widespread vaccination, there is a global resurgence of pertussis and a large increase in cases reported after the COVID-19 pandemic (*2*). The pertussis resurgence is likely multifactorial, including declining vaccination coverage, improved diagnostic capacity, waning immunity, and the evolutionary adaptation of *B. pertussis* bacteria (*3*).

Macrolide antimicrobial drugs are the first-line pertussis treatment because of their effectiveness in reducing bacterial carriage and transmission, curtailing the duration of symptoms, and lowering the risk for infant death (*4*). However, macrolide-resistant *B. pertussis* (MRBP), primarily associated with the A2047G mutation in 23S rRNA genes, has emerged globally (*5*). The highest prevalence of MRBP has been reported in China (*6*), whereas reports of MRBP from Latin America remain scarce (*7*). 

In 2025, the Peruvian Ministry of Health reported the largest pertussis outbreak in decades in the country. Although precisely defining the onset of the outbreak is challenging, surveillance data suggest that transmission intensified in 2024. National incidence increased from 3.9 cases/1 million inhabitants in 2023 to 7.4 cases/1 million inhabitants in 2024 and reached 57.5 cases/1 million inhabitants by August 2025 (*8*). A cumulative total of 4,976 cases (Appendix Figure) and 76 deaths were recorded by the end of 2025. The effects were concentrated in the Loreto region, which accounted for 3,922 cases and 57 deaths, disproportionately affecting Indigenous communities in Datem del Marañón, where pertussis vaccine coverage remains suboptimal (*9*,*10*). 

Peru’s national immunization schedule includes 3 primary doses of the pentavalent diphtheria–tetanus–whole cell pertussis–hepatitis B–*Hemophilus influenzae* type b vaccine (DTwP-HepB-Hib) at 2, 4, and 6 months of age, followed by DTwP boosters at 18 months and 4 years of age, and tetanus–diphtheria–acellular pertussis vaccination during pregnancy, recommended at 20–36 weeks of gestation. To determine *B. pertussis* transmission dynamics in Peru, we detected and characterized MRBP from *B. pertussis*–positive swab samples. 

## The Study

In early 2025, we screened for MRBP in *B. pertussis*–positive nasopharyngeal swab samples from 68 patients (*11*) (median 3 months of age; range 1 month–39 years of age) by using direct PCR (Appendix Table 1) targeting the A2047G mutation in the 23S rRNA genes. We detected the A2047G mutation in 21 (31%) of 68 samples. Patients infected with MRBP were significantly younger (median 3.6 vs 36.1 months of age; p = 0.007) than patients infected with macrolide-susceptible *B. pertussis* strains. However, we observed no significant differences in patient sex or vaccination status between the 2 groups (Appendix Table 2). Most MRBP infections were in infants <6 months of age (62%). Among the 16 patients with available treatment information, 14 (88%) received azithromycin. MRBP cases were distributed across multiple regions, including Lima (n = 11), Cusco (n = 4), Arequipa (n = 3), Ancash (n = 1), Loreto (n = 1), and Puno (n = 1) (Table; Figure 1; Appendix Table 2). 

**Table:** Characteristics of patients with culture-confirmed *Bordetella pertussis* carrying the macrolide-resistant A2047G mutation in the 23S rRNA gene, Peru, 2025

Sample no.	Year of collection	Age/sex	Region, Province	Vaccine status†	Maternal Tdap‡	Antimicrobial drug treatment	Hospitalized
tf518-24*	2024	9 mo/F	Loreto, Datem del Marañón	2 doses	Unknown	No	Unknown
tf048-25	2025	1.6 y/M	Puno, Melgar	Unknown	No	No	Yes
tf098-25	2025	3 mo/F	Lima, Callao	0	No	Azithromycin	Yes
tf120-25	2025	39.5 y/F	Lima, Callao	Unknown	No	Unknown	Unknown
tf232-25	2025	16 y/M	Cusco, Cusco	1 dose	No	Azithromycin	Unknown
tf303-25	2025	1 mo/F	Arequipa, Arequipa	0	Unknown	Azithromycin, ampicillin, and amikacin	Yes
tf353-25	2025	7 mo/M	Cusco, Cusco	3 doses	Yes	Azithromycin	Unknown

*This isolate, collected in late 2024, was considered part of the early phase of the 2025 outbreak. †Number of diphtheria–tetanus–whole cell pertussis–hepatitis B–*Hemophilus influenzae* type b vaccine doses received before illness onset. ‡Tdap (tetanus–diphtheria–acellular pertussis) vaccination during pregnancy in the patient’s mother.

**Figure 1:**
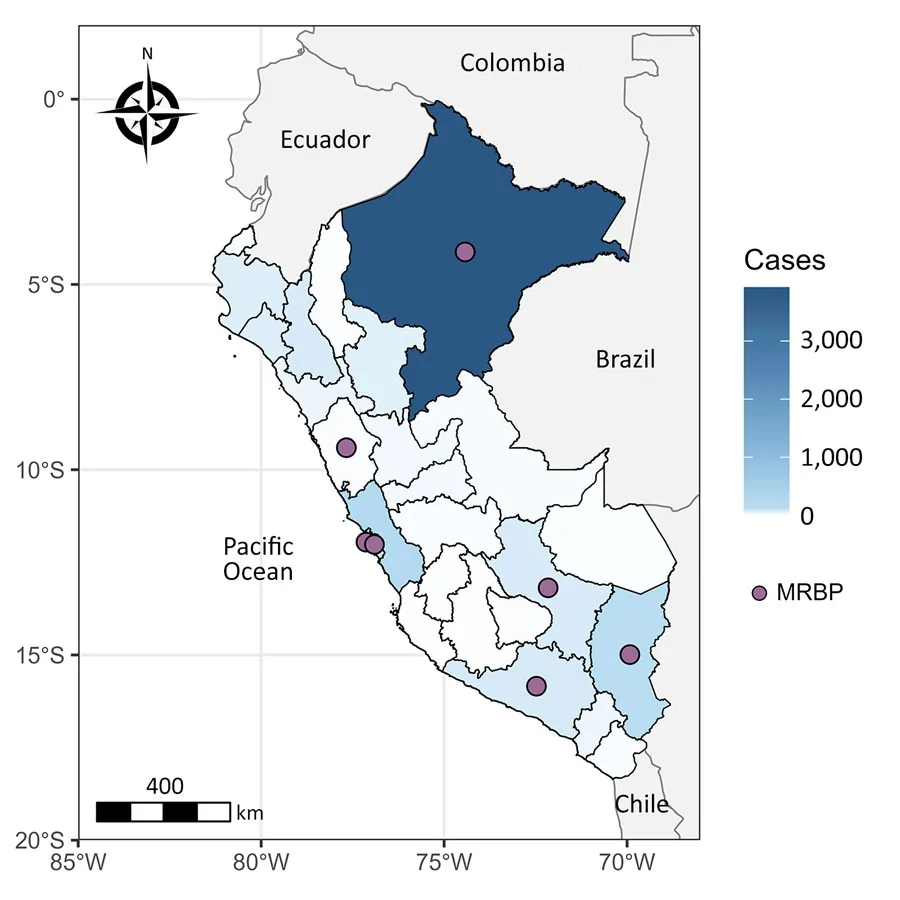
Geographic distribution of confirmed pertussis cases and MRBP cases in Peru, 2025. Regional shading shows cumulative confirmed pertussis cases since epidemiologic week 53 of 2025; darker blue indicates higher case counts. Purple circles indicate regions where patients carrying *B. pertussis* strains with the macrolide resistance–associated A2047G mutation were detected. MRBP, macrolide-resistant *Bordetella pertussis*.

From the 68 *B. pertussis–*positive samples, we obtained 7 culture-positive *B. pertussis* isolates, all of which were MRBP: 6 collected in 2025 and 1 in late 2024 (isolate no. tf518-24). We performed phenotypic susceptibility testing by using established methods (*12*). All 7 isolates were resistant to all antimicrobial drugs tested, with no zones of inhibition for erythromycin or azithromycin. 

We performed whole-genome sequencing on all 7 MRBP strains (Appendix). All sequenced isolates were assigned to sequence type 2 (ST2) and clonal complex ST2. Six isolates shared the virulence-associated allelic profile *ptxP3/ptxA1/ptxB1/ptxC4/fhaB1/fim2-1/fim3-1*, whereas 1 isolate (tf098–25) carried the closely related *ptxP29* promoter allele but retained the remaining alleles of this profile. We identified the *prn150* allele in all sequenced isolates (Appendix), which is a genetic marker of the emerging MR-MT28 lineage (*8*). We did not identify any inactivating mutations in the *ptx* operon or *fhaB*, but 3 isolates (tf518-24, tf120-25, and tf098-25) had IS481-mediated *prn* disruption, consistent with a pertactin-deficient genotype (Appendix).

Expanded whole-genome single-nucleotide polymorphism phylogenetic analysis incorporating globally representative MRBP genomes placed all isolates from Peru within the recently emerged MR-MT28 lineage. That expanded analysis also detected previously identified genetic markers, including the A2047G mutation, the *prn150* allele, and the *BP0685* G1959A substitution (*8*). The 7 MRBP isolates from Peru did not form a single monophyletic cluster. Three isolates (tf518-24, tf120-25, and tf098-25) clustered within the phylogenetic group 1 (PG1) sublineage and had IS481-mediated *prn* disruption, whereas the remaining 4 isolates clustered with the MR-MT28-other group and retained an intact *prn* coding sequence despite carrying the *prn150* allele (Figure 2; Appendix). 

**Figure 2:**
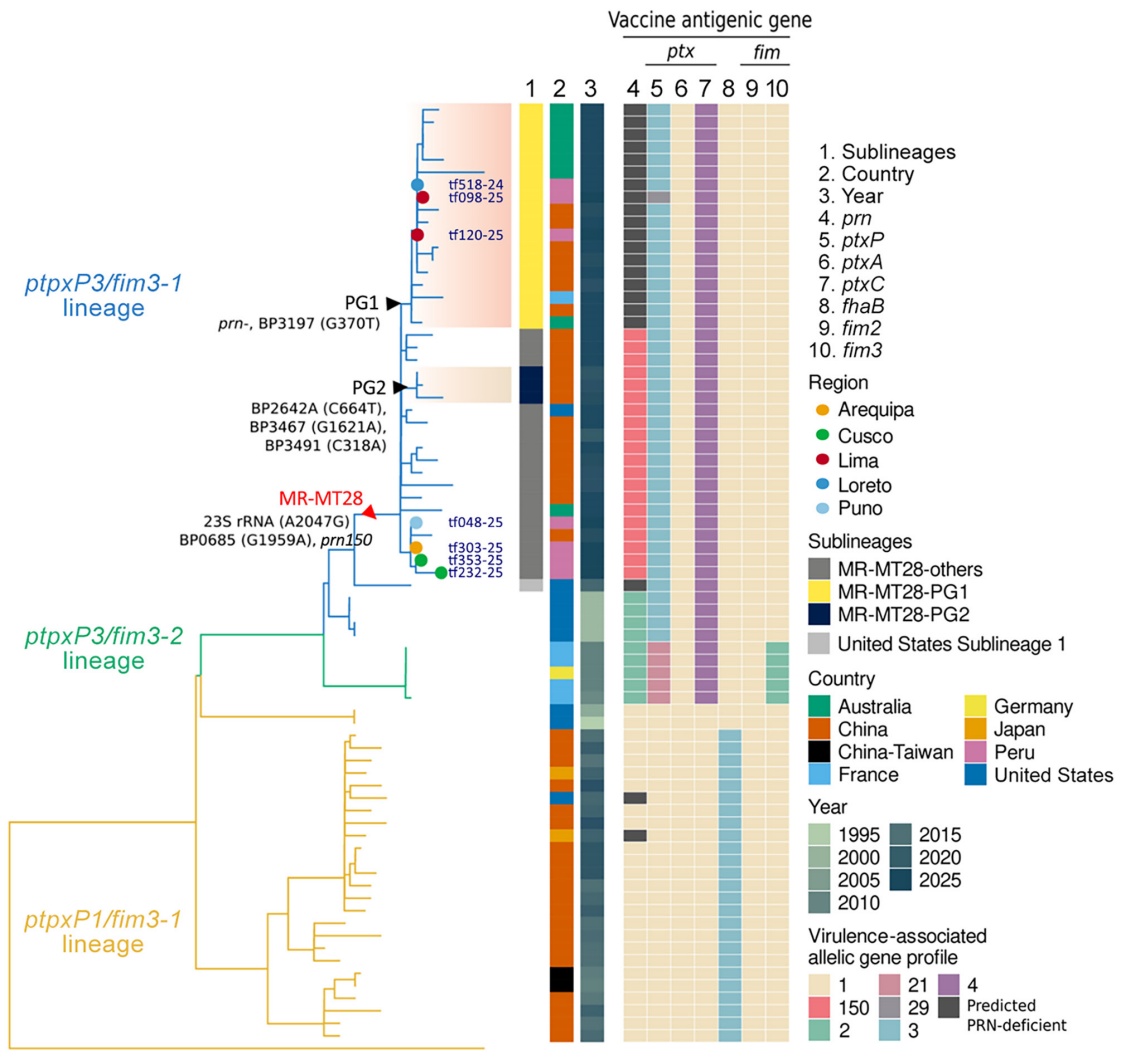
Maximum-likelihood phylogenetic tree of *Bordetella pertussis* isolates on the basis of genome-wide single-nucleotide polymorphism analysis, showing macrolide-resistant isolates from Peru within the emerging MR-MT28 lineage (red text) among globally distributed strains. Tohama I (NC_002929) was used as the reference genome. PRN, pertactin.

## Conclusions

We described the emergence of MRBP during the ongoing 2025 pertussis outbreak in Peru and documented circulation of the emerging MR-MT28 lineage in Latin America. Genomic analyses identified >2 genetically distinct MR-MT28 sublineages, including PG1-associated pertactin-deficient isolates, consistent with the ongoing international diversification and dissemination of this lineage.

Consistent with recent global genomic analyses, the close phylogenetic relationship between the 7 isolates from Peru and contemporary MR-MT28 strains is consistent with the ongoing international dissemination and diversification of this lineage (*5*,*13*). The presence of 2 genetically distinct MR-MT28 groups circulating in Peru suggests multiple international introductions of MRBP into Peru, rather than a single transmission event.

Pertactin-deficient isolates have predominantly been reported in countries using acellular pertussis vaccines, where loss of pertactin is considered an adaptive response to vaccine-induced immune pressure (*5*,*13*). Although Peru continues to use a whole-cell pertussis vaccine, 3 PG1 sublineage isolates had IS481-mediated *prn* disruption, suggesting introduction of an established pertactin-deficient MR-MT28 sublineage, rather than independent emergence under local vaccine-driven selection. Of note, 1 of those isolates (tf518-24) was recovered from an infant who received 2 doses of the pentavalent vaccine, indicating that a pertactin-deficient MR-MT28 strain was identified in a partially vaccinated infant.

We found that most patients with MRBP received azithromycin for treatment, which raises concerns about the effectiveness of macrolides as first-line therapy in Peru. Although *B. pertussis* has remained susceptible to sulfamethoxazole/trimethoprim in vitro (*14*) and possibly to other antimicrobial drugs (*15*), clinical evidence supporting alternative regimens is limited, underscoring the need for urgent clinical and epidemiologic studies.

A key limitation of our study is the lack of systematically collected clinical data linked to genomic findings within the current surveillance system, which precludes assessment of differences in disease severity and outcomes between macrolide-resistant and macrolide-susceptible *B. pertussis* infections during this outbreak. Addressing that gap will be critical to define the clinical impact of macrolide resistance and to inform evidence-based treatment strategies in high-prevalence settings.

 Our findings underscore the urgent need to strengthen genomic surveillance and diagnostic capacity for *B. pertussis* to enable early detection of resistant strains and inform timely prevention and control strategies. In addition, reassessing empiric treatment recommendations and prioritizing prospective studies to evaluate the clinical effect of macrolide antimicrobial drug resistance will be critical to guide evidence-based public health responses.

Appendix Additional information about emergence of macrolide-resistant *Bordetella pertussis*, Peru, 2025.
