# Supplementary material for: Emergence of Macrolide-Resistant Bordetella pertussis, Peru, 2025
Source: Emerg Infect Dis. 2026 Oct;32(10):1702–6. doi: 10.3201/eid3210.251477 (PMC13632835; doi:10.3201/eid3210.251477)
Supplement: Supplementary file 1 [file 25-1477-Techapp-s1.pdf]

Article DOI: https://doi.org/10.3201/eid3210.251477

EID cannot ensure accessibility for supplementary materials supplied by authors. Readers who have difficulty accessing supplementary content should contact the authors for assistance.

Emergence of Macrolide-Resistant *Bordetella pertussis*, Peru, 2025

Appendix

Erythromycin Resistance PCR (ER-PCR) for Detection of the A2047G Mutation

A conventional erythromycin resistance PCR (ER-PCR), adapted from Wang et al. (*1,2*), was used to detect the A2047G mutation in the *23S rRNA* gene of *Bordetella pertussis*. To discriminate between the mutant (A2047G) and wild-type alleles, two separate allele-specific PCR assays were performed using a common external primer pair (FP and RP) and either a mutant-specific primer (MP) or a wild-type-specific primer (WP). Primer sequences are listed in Appendix Table 1.

PCR amplification was performed in 20-µL reactions containing 1× HotStarTaq Master Mix (Qiagen), 8% (vol/vol) dimethyl sulfoxide (DMSO), 0.5 µM each of the forward and reverse primers, 0.5 µM of either the mutant-specific or wild-type-specific primer, and 2 µL of DNA template. PCR conditions consisted of an initial enzyme activation at 95°C for 15 min, followed by 35 cycles of 94°C for 60 s, 60°C for 30 s, and 72°C for 30 s, with a final extension at 72°C for 10 min. The external primer pair generated a 286-bp control amplicon, whereas the allele-specific primer generated a 121-bp amplicon specific for either the mutant or wild-type allele. PCR products were resolved by electrophoresis on 2% agarose gels and compared with a 100-bp DNA ladder.

Whole-Genome Sequencing, Assembly, and Quality Assessment

Genomic DNA from all isolates was sequenced on an Illumina MiSeq platform using a 500-cycle reagent kit (2 × 250-bp paired-end reads). Libraries were prepared with the Nextera XT DNA Library Preparation Kit and Nextera XT Index Kit v2 Set A. Raw reads were quality

filtered and adaptor trimmed using Trimmomatic v0.39 (*3*) before de novo assembly with SPAdes v3.13.0 (*4*). Assembly quality was assessed using CheckM v1.2.0 (*5*), and only genomes with >95% completeness and <5% contamination were retained for downstream analyses.

WGS-Based MLST and Vaccine antigen–based allelic profiling

Whole-genome sequence (WGS)-based multilocus sequence typing (MLST), based on the allelic profiles of the seven housekeeping genes (*adk, fumC, glyA, tyrB, icd, pepA,* and *pgm*), and allelic profiling of acellular vaccine antigen genes (*ptxA, ptxB, ptxC, ptxD, ptxE, ptxP, fim2, fim3, fhaB,* and *prn*) were performed using mlst v2.23.0 (https://github.com/tseemann/mlst). Sequence types (STs), clonal complexes (CCs), and allele assignments were determined using the BIGSdb-Pasteur *Bordetella* database (*6*).

For uncommon *ptxP* alleles, assembled *ptxP* promoter sequences were additionally compared with the *B. pertussis* Tohama I reference sequence to confirm allele assignment. According to the BIGSdb-Pasteur *Bordetella* allele nomenclature, *ptxP29* differs from *ptxP1* by two nucleotide substitutions (G109A and T166C) and from *ptxP3* by a single T166C substitution.

Sequence-Based *prn150* Allele Assignment

Because the *prn150* allele is not currently represented in the BIGSdb-Pasteur *Bordetella* database, we performed an additional sequence-based analysis. Genome assemblies were analyzed by BLASTn (*7*) against the published *prn150* sequence of *B. pertussis* strain YW58 (GenBank accession no. CP178779.1). Although complete *prn* gene sequences were recovered for only two isolates, the assembled contigs containing the *prn* gene were identical to the reference *prn150* sequence in all seven isolates. In addition, the C531T substitution, previously described as a defining polymorphism of the *prn150* allele (*8*), was detected in all isolates, supporting assignment of the *prn150* allele.

Pertussis toxin operon (*ptxA–E*) and *ptxP* promoter analysis

The genomic integrity of the pertussis toxin operon (*ptxA–E*) was assessed in all seven isolates by comparing assembled sequences against the corresponding genes of the *B. pertussis*

Tohama I reference genome. BLASTn analysis showed that the complete *ptx* operon was contained within a single contig in all isolates, enabling full-length sequence inspection. No premature stop codons, frameshift mutations, large deletions, or other inactivating mutations were detected in any of the *ptxA–E* genes.

Relative to the Tohama I reference sequence, all isolates carried a G684A substitution in *ptxA*, resulting in a predicted amino acid change from methionine to isoleucine, and a G133A substitution in *ptxB*, resulting in a glycine-to-serine substitution. In addition, a synonymous C181T substitution was identified in *ptxC*, which did not alter the encoded amino acid sequence. No nucleotide differences were detected in *ptxD* or *ptxE*.

The *ptxP* promoter region was also analyzed by BLASTn (*7*). Six of the seven isolates displayed a sequence identical to the *ptxP3* reference allele (KM458469.1), whereas a single isolate (tf098–25) carried a T234C substitution and was assigned to the *ptxP29* allele according to the BIGSdb-Pasteur *Bordetella* allele database. Overall, no genomic evidence of disruption or inactivation of the pertussis toxin operon was observed among the analyzed isolates.

Analysis of *fhaB* gene integrity

BLASTn analysis was performed using the *fhaB* gene reference (Gene ID: 69601663). The *fhaB* locus was recovered in multiple contigs (4–5 contigs) across all analyzed isolates, with assembly breakpoints consistently occurring within repetitive regions of the gene. Inspection of the assembled contigs did not reveal premature stop codons or frameshift mutations within the recovered coding regions. In addition, the 5′ and 3′ coding regions, including the canonical start and stop codons, were present and conserved relative to the reference sequence. However, due to the highly repetitive nature of the *fhaB* locus and limitations associated with short-read Illumina sequencing, complete gene reconstruction was not achieved. Therefore, while no evidence of inactivating mutations was detected in the assembled regions, mutations within unresolved repetitive regions cannot be excluded.

Detection of IS481-mediated *prn* gene disruption

IS481-mediated disruption of the *prn* gene was investigated using ISMapper v2.0 *(9*) to analyze raw sequencing reads. Putative insertion sites were further evaluated by mapping reads

against the *B. pertussis* SHBP943 reference genome (GenBank accession no. CP163268.1), which contains a characterized IS481 insertion within *prn*. Reads were aligned using BWA-MEM v0.7.18 (*10*) and inspected with SAMtools v1.20 (*11*) to support IS481-associated disruption of *prn*, identifying insertions in isolates tf518–24, tf120–25, and tf098–25.

Detection of the A2047G mutation associated with macrolide resistance

Macrolide resistance-associated mutations were investigated by mapping raw sequencing reads against the *B. pertussis* Tohama I reference genome using BWA-MEM v0.7.18 and inspected with SAMtools v1.20 (*11*). Alignments were manually inspected using Integrative Genomics Viewer (IGV) v2.19.4 (*12*) to determine the presence and copy number of the A2047G mutation in the *23S rRNA* genes.

SNP-based phylogenetic analysis

To improve the global phylogenetic context, we expanded our analysis by incorporating 65 additional publicly available macrolide-resistant *B. pertussis* (MRBP) genomes representing isolates from People's Republic of China, Australia, France, the United States, Japan, Germany, and Taiwan, based on the dataset reported by Zhang et al. (*8*). A whole-genome SNP-based phylogenetic analysis (520 core-genome SNPs relative to the Tohama I reference genome) was then performed including the seven Peruvian MRBP isolates. Variant calling and core-genome SNP alignment were performed using Snippy v4.6.0 (https://github.com/tseemann/snippy), and SNP positions were extracted using SNP-sites v2.5.1 (*13*). Recombinant regions were identified and removed using Gubbins v3.3.1 (*14*) before maximum-likelihood phylogenetic reconstruction with IQ-TREE2 v2.3.0 (*15*). The best-fit nucleotide substitution model was selected using ModelFinder (TVMe+ASC), and branch support was assessed with 10,000 bootstrap replicates.

Statistical Analysis

Continuous variables were summarized as medians and interquartile ranges (IQRs), whereas categorical variables were summarized as frequencies and percentages. Differences in age between patients with macrolide-resistant and macrolide-susceptible *B. pertussis* infection were assessed using the Mann–Whitney U test. Categorical variables were compared using the

Fisher–Freeman–Halton exact test, except for sex, which was analyzed using the Pearson χ^2 test with Yates continuity correction. All statistical tests were two-sided, and p values <0.05 were considered statistically significant. Statistical analyses were performed in R version 4.5.1.

References

1. Wang Z, Han R, Liu Y, Du Q, Liu J, Ma C, et al. Direct detection of erythromycin-resistant *Bordetella pertussis* in clinical specimens by PCR. J Clin Microbiol. 2015;53:3418–22. PubMed https://doi.org/10.1128/JCM.01499-15
2. Wang Z, Cui Z, Li Y, Hou T, Liu X, Xi Y, et al. High prevalence of erythromycin-resistant *Bordetella pertussis* in Xi'an, China. Clin Microbiol Infect. 2014;20:O825–30. PubMed https://doi.org/10.1111/1469-0691.12671
3. Bolger AM, Lohse M, Usadel B. Trimmomatic: a flexible trimmer for Illumina sequence data. Bioinformatics. 2014;30:2114–20. PubMed https://doi.org/10.1093/bioinformatics/btu170
4. Prjibelski A, Antipov D, Meleshko D, Lapidus A, Korobeynikov A. Using SPAdes de novo assembler. Curr Protoc Bioinformatics. 2020;70:e102. PubMed https://doi.org/10.1002/cpbi.102
5. Parks DH, Imelfort M, Skennerton CT, Hugenholtz P, Tyson GW. CheckM: assessing the quality of microbial genomes recovered from isolates, single cells, and metagenomes. Genome Res. 2015;25:1043–55. PubMed https://doi.org/10.1101/gr.186072.114
6. Jolley KA, Bray JE, Maiden MCJ. Open-access bacterial population genomics: BIGSdb software, the PubMLST.org website and their applications. Wellcome Open Res. 2018;3:124. PubMed https://doi.org/10.12688/wellcomeopenres.14826.1
7. Altschul SF, Gish W, Miller W, Myers EW, Lipman DJ. Basic local alignment search tool. J Mol Biol. 1990;215:403–10. PubMed https://doi.org/10.1016/S0022-2836(05)80360-2
8. Zhang H, Kang Z, Zhang Y, Yang Y, Li H, Wang N, et al. Evolutionary dynamics and global spread of macrolide-resistant *Bordetella pertussis* during the post-pandemic pertussis resurgence. J Infect. 2026;92:106718. PubMed https://doi.org/10.1016/j.jinf.2026.106718
9. Hawkey J, Hamidian M, Wick RR, Edwards DJ, Billman-Jacobe H, Hall RM, et al. ISMapper: identifying transposase insertion sites in bacterial genomes from short read sequence data. BMC Genomics. 2015;16:667. PubMed https://doi.org/10.1186/s12864-015-1860-2
10. Li H, Durbin R. Fast and accurate short read alignment with Burrows-Wheeler transform. Bioinformatics. 2009;25:1754–60. PubMed https://doi.org/10.1093/bioinformatics/btp324

11. Danecek P, Bonfield JK, Liddle J, Marshall J, Ohan V, Pollard MO, et al. Twelve years of SAMtools and BCFtools. Gigascience. 2021;10:giab008. PubMed https://doi.org/10.1093/gigascience/giab008

12. Robinson JT, Thorvaldsdóttir H, Winckler W, Guttman M, Lander ES, Getz G, et al. Integrative genomics viewer. Nat Biotechnol. 2011;29:24–6. PubMed https://doi.org/10.1038/nbt.1754

13. Page AJ, Taylor B, Delaney AJ, Soares J, Seemann T, Keane JA, et al. *SNP-sites*: rapid efficient extraction of SNPs from multi-FASTA alignments. Microb Genom. 2016;2:e000056. PubMed https://doi.org/10.1099/mgen.0.000056

14. Croucher NJ, Page AJ, Connor TR, Delaney AJ, Keane JA, Bentley SD, et al. Rapid phylogenetic analysis of large samples of recombinant bacterial whole genome sequences using Gubbins. Nucleic Acids Res. 2015;43:e15. PubMed https://doi.org/10.1093/nar/gku1196

15. Minh BQ, Schmidt HA, Chernomor O, Schrempf D, Woodhams MD, von Haeseler A, et al. IQ-TREE 2: new models and efficient methods for phylogenetic inference in the genomic era. Mol Biol Evol. 2020;37:1530–4. PubMed https://doi.org/10.1093/molbev/msaa015

Appendix Table 1. Primers used for erythromycin resistance

Primer	Sequence (5′–3′)	Reference
Forward Primer (FP)	GTGATGGGGTGCAAGCTCTT	(*1*)
Reverse Primer (RP)	TCTGGCGACTCGAGTTCTGC	(*2*)
Mutant type primer (MP)	ATCTACCCGCGGCTAGACAGG	(*1*)
Wild type primer (WP)	ATCTACCCGCGGCTAGACAGA	(*1*)

Appendix Table 2. Characteristics of patients with macrolide-resistant and macrolide-susceptible *Bordetella pertussis* infections identified through March 2025 during the early phase of the pertussis outbreak in Peru*

Characteristic	Macrolide-resistant (n = 21)	Macrolide-susceptible (n = 47)	Total (n = 68)	*P* value
Age, months, median (IQR)	3.6 (2.4–12.9)	36.1 (3.8–205.4)	14.2 (3.4–140.9)	0.007§
Age group				0.233¶
<2 mo	4 (19.0%)	3 (6.4%)	7 (10.3%)	
2–<6 mo	9 (42.9%)	12 (25.5%)	21 (30.9%)	
6–<12 mo	2 (9.5%)	3 (6.4%)	5 (7.4%)	
1–4 y	3 (14.3%)	12 (25.5%)	15 (22.1%)	
5–11 y	1 (4.8%)	2 (4.3%)	3 (4.4%)	
12–29 y	1 (4.8%)	10 (21.3%)	11 (16.2%)	
≥30 y	1 (4.8%)	5 (10.6%)	6 (8.8%)	
Sex				0.969#
Female	12 (57.1%)	25 (53.2%)	37 (54.4%)	
Male	9 (42.9%)	22 (46.8%)	31 (45.6%)	
Vaccination status†				0.721¶
No doses	7 (50.0%)	7 (46.7%)	14 (48.3%)	
1 dose	5 (35.7%)	5 (33.3%)	10 (34.5%)	
2 doses	1 (7.1%)	0 (0.0%)	1 (3.4%)	
3 doses	1 (7.1%)	3 (20.0%)	4 (13.8%)	
Region				<0.001¶
Ancash	1 (4.8%)	0 (0.0%)	1 (1.5%)	
Arequipa	3 (14.3%)	0 (0.0%)	3 (4.4%)	
Cajamarca	0 (0.0%)	2 (4.3%)	2 (2.9%)	
Cusco	4 (19.0%)	1 (2.1%)	5 (7.4%)	
Lima	11 (52.4%)	9 (19.1%)	20 (29.4%)	
Loreto	1 (4.8%)	29 (61.7%)	30 (44.1%)	
Piura	0 (0.0%)	1 (2.1%)	1 (1.5%)	
Puno	1 (4.8%)	0 (0.0%)	1 (1.5%)	
Tacna	0 (0.0%)	5 (10.6%)	5 (7.4%)	
Antimicrobial treatment‡				0.840¶
Azithromycin	14 (87.5%)	26 (83.9%)	40 (85.1%)	
Other macrolides	1 (6.3%)	1 (3.2%)	2 (4.3%)	
Non-macrolides	1 (6.3%)	4 (12.9%)	5 (10.6%)	

*Values are no. (%) unless indicated. IQR, interquartile range.
†Vaccination status available for 29 patients.
‡Antimicrobial treatment available for 47 patients.
§Mann–Whitney U test.
¶Fisher–Freeman–Halton exact test.
# Pearson χ^2 test with Yates continuity correction.

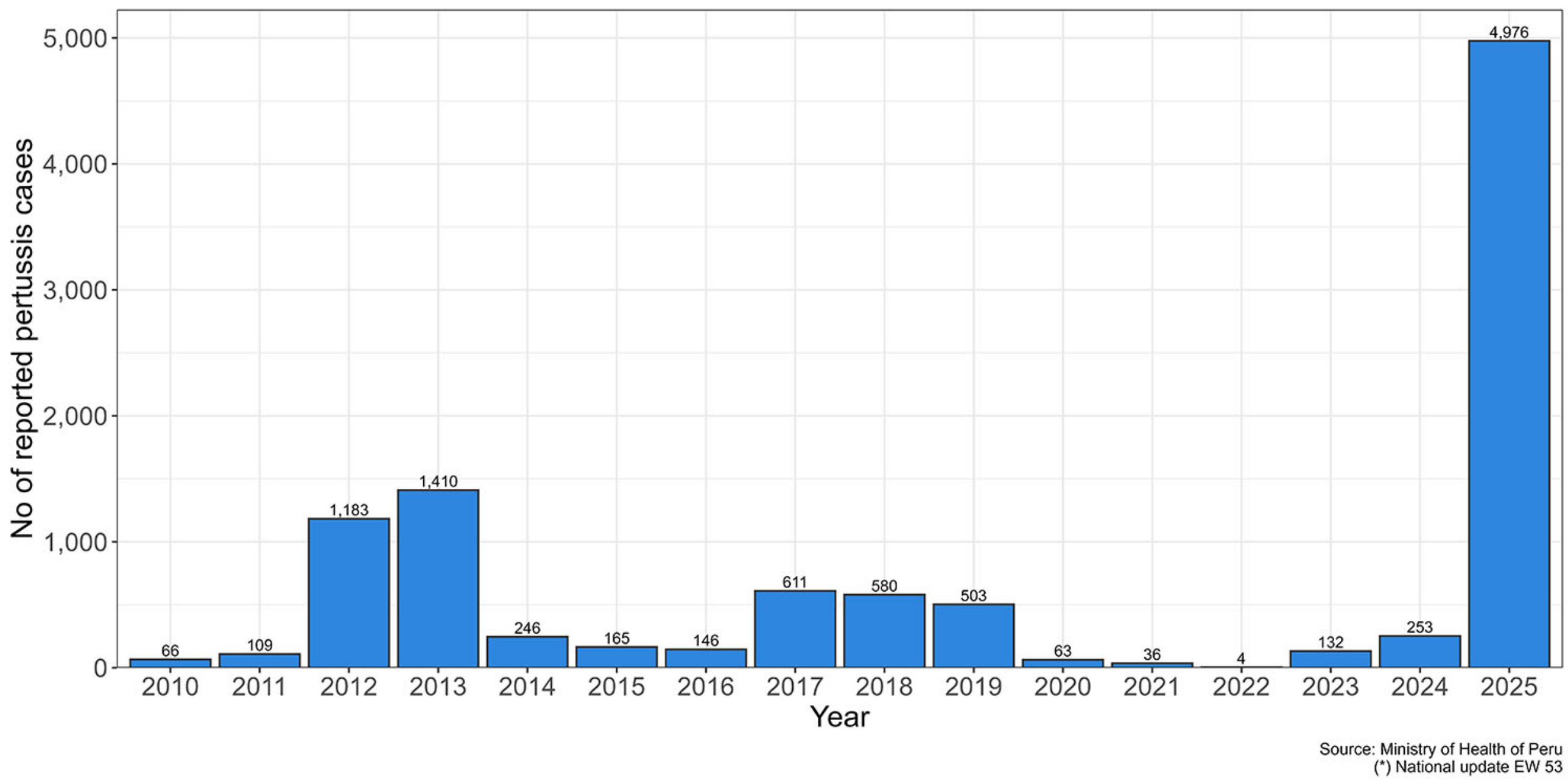

Appendix Figure. Reported pertussis cases in Peru, 2010–2025. Data were obtained from the Ministry of Health of Peru. A sharp increase is observed in 2025, with 4,976 cases reported as of epidemiologic week 53, exceeding annual totals reported in the previous 15 years.
